# Statistical analysis on hollow and core-shell structured vanadium oxide microspheres as cathode materials for Lithium ion batteries

**DOI:** 10.1016/j.dib.2018.01.105

**Published:** 2018-02-05

**Authors:** Xing Liang, Guohua Gao, Guangming Wu

**Affiliations:** Shanghai Key Laboratory of Special Artificial Microstructure Materials and Technology, School of Physics Science and Engineering, Tongji University, Shanghai 200092, China

**Keywords:** Adsorption-desorption isotherm, Pore size distribution, SEM images, TEM images

## Abstract

In this data, the statistical analyses of vanadium oxide microspheres cathode materials are presented for the research article entitled “Statistical analyses on hollow and core-shell structured vanadium oxides microspheres as cathode materials for Lithium ion batteries” (Liang et al., 2017) [1]. This article shows the statistical analyses on N_2_ adsorption-desorption isotherm and morphology vanadium oxide microspheres as cathode materials for LIBs.

**Specifications Table**

**Table:** 

Subject area	*Electrochemistry*
More specific subject area	*Lithium ion batteries*
Type of data	*Figure*
How data was acquired	*SEM, Xtended Pressure Sorption Analyzer (ASAP 2020, Micromeritics, USA)*
Data format	*analyzed*
Experimental factors	*Brunauer−Emmett−Teller (BET) absorption and the pore size distribution were measured on an Xtended Pressure Sorption Analyzer (ASAP 2020, Micromeritics, USA) at 77 K. The specific surface area and pore diameter distribution of the samples were analyzed by BET and Barrett-Joyner-Halenda (BJH) methods.*
	*The FESEM images, TEM images of the microspheres were characterized by field emission scanning electron microscopy (FESEM, S-4800, Hitachi, Japan) and transmission electron microscopy (TEM, JEM-2100F, Jeol, Japan)*
Experimental features	*The specific surface area and pore size distribution were determined by Xtended Pressure Sorption Analyzer*
Data source location	*Shanghai, China*
Data accessibility	*The data are with this article*
Related research article	*Xing Liang, Guohua Gao, Guangming Wu, Huiyu Yang*
	*Synthesis and characterization of novel hierarchical starfish-like*
	*vanadium oxide and their electrochemical performance. Electrochimica Acta, 188 (2017), 625–635.*

**Value of the data**

• The data presents specific surface area and pore size distribution of vanadium oxides hollow microspheres.
• The SEM images of the solvothermal reaction products using different volume ratio of isopropanol/ethylene glycol was characterized by field emission scanning electron microscopy (FESEM, S-4800, Hitachi, Japan) and showed an appropriate amount of ethylene glycol is important for the formation of uniform microspheres structure.
• The FESEM images and TEM images of the microspheres prepared with different solvothermal reaction time were characterized by field emission scanning electron microscopy (FESEM, S-4800, Hitachi, Japan) and transmission electron microscopy (TEM, JEM-2100F, Jeol, Japan) and showed the morphology of core-shell microspheres.

## 1 Data

**Fig. 1:**
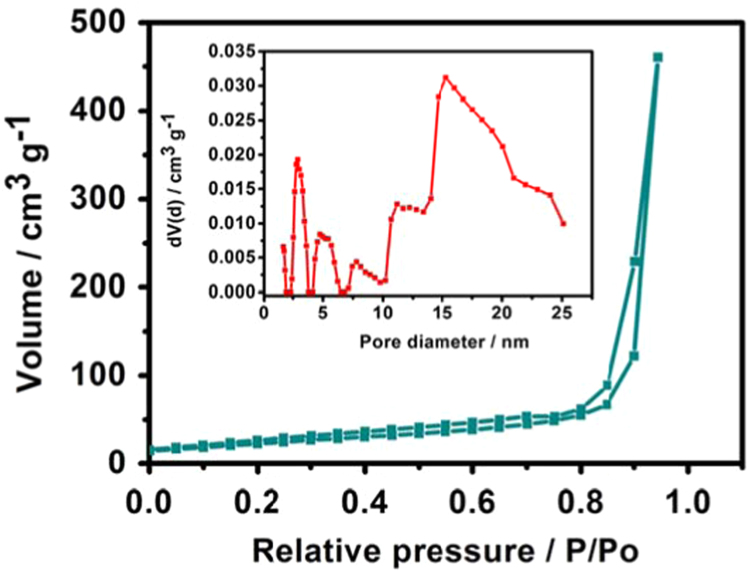
N_2_ adsorption-desorption isotherm and the corresponding pore size distribution curves (inset) of vanadium oxides hollow microspheres.

**Fig. 2:**
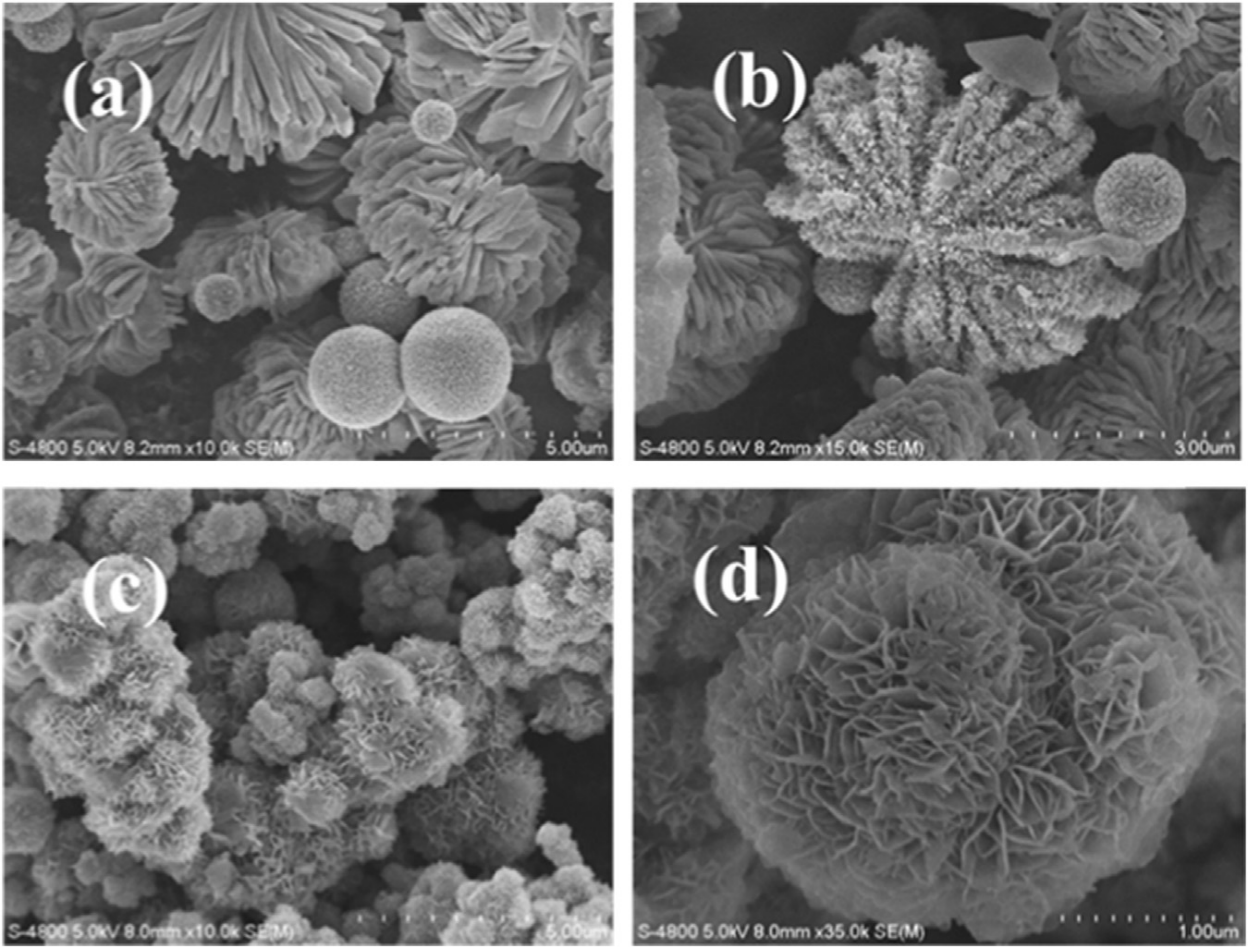
FESEM images of the solvothermal reaction products using different volume ratio of isopropanol/ethylene glycol: (a and b) 30 mL of isopropanol and 0 ml of ethylene glycol, (b) 10 mL of isopropanol and 20 ml of ethylene glycol.

**Fig. 3:**
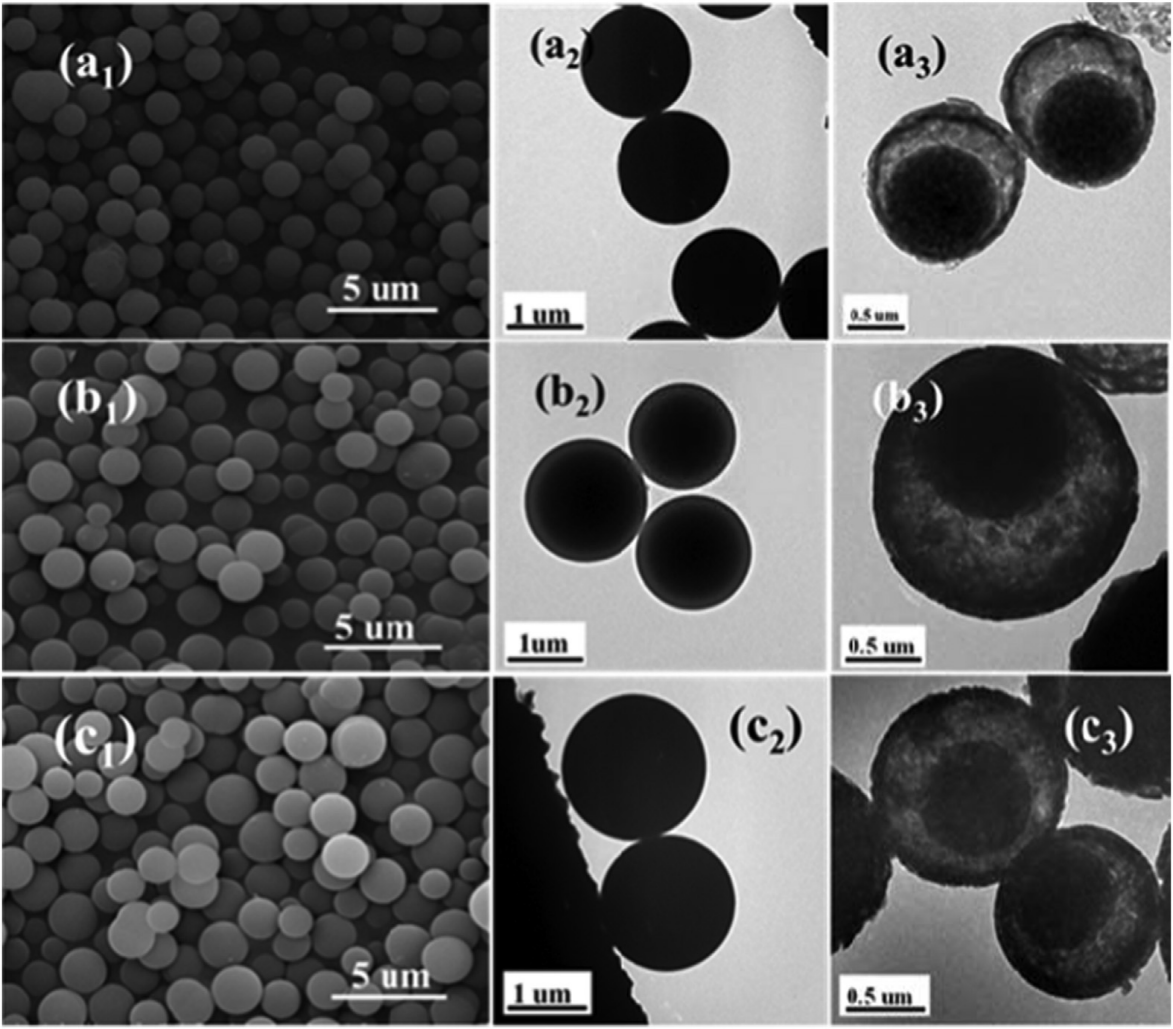
FESEM images, TEM images of the microspheres prepared with different solvothermal reaction time: (a_1_–a_3_) 5 h, (b_1_–b_3_) 7 h, (c_1_–c_3_) 48 h. (the images in the first two columns represent the as-prepared precursors, the images in the third column represent the calcined microspheres.).

The Fig. 1 was acquired from Origin 85 software by plotting the N_2_ adsorption-desorption isotherm and the corresponding pore size distribution curves (inset) data. The SEM image in Fig. 2 was acquired from field-emission scanning electron microscope (FE-SEM, S-4800). The FESEM images and TEM images of the microspheres prepared with different solvothermal reaction time (5 h, 7 h and 48 h) in Fig. 3 were characterized by field emission scanning electron microscopy (FESEM, S-4800) and transmission electron microscopy (TEM, JEM-2100F) [1].

## 2 Experimental design, materials and methods

### 2.1 Measurements

Brunauer−Emmett−Teller (BET) absorption and the pore size distribution were measured on an Xtended Pressure Sorption Analyzer (ASAP 2020, Micromeritics, USA) at 77 K. The specific surface area and pore diameter distribution of the samples were analyzed by BET and Barrett-Joyner-Halenda (BJH) methods. The morphology of the sample was determined using field emission scanning electron microscopy (FESEM, S-4800, Hitachi, Japan) and transmission electron microscopy (TEM, JEM-2100F, Jeol, Japan).
